# Unlocking the Transcriptional Control of *NCAPG* in Bovine Myoblasts: CREB1 and MYOD1 as Key Players

**DOI:** 10.3390/ijms25052506

**Published:** 2024-02-21

**Authors:** Zongchang Chen, Jingsheng Li, Yanbin Bai, Zhanxin Liu, Yali Wei, Dashan Guo, Xue Jia, Bingang Shi, Xiaolan Zhang, Zhidong Zhao, Jiang Hu, Xiangmin Han, Jiqing Wang, Xiu Liu, Shaobin Li, Fangfang Zhao

**Affiliations:** Gansu Key Laboratory of Herbivorous Animal Biotechnology, College of Animal Science and Technology, Gansu Agricultural University, Lanzhou 730070, China; chenzongc@st.gsau.edu.cn (Z.C.); lijs@st.gsau.edu.cn (J.L.); baiyb@st.gsau.edu.cn (Y.B.); liuzx@st.gsau.edu.cn (Z.L.); weiyl@st.gsau.edu.cn (Y.W.); guods@st.gsau.edu.cn (D.G.); jiax@st.gsau.edu.cn (X.J.); shibg@gsau.edu.cn (B.S.); zhangxl@gsau.edu.cn (X.Z.); huj@gsau.edu.cn (J.H.); 13893362306@163.com (X.H.); wangjq@gsau.edu.cn (J.W.); liux@gsau.edu.cn (X.L.); lisb@gsau.edu.cn (S.L.); zhaoff@gsau.edu.cn (F.Z.)

**Keywords:** promoter, transcriptional regulation, *NCAPG*, bovine, myoblast

## Abstract

Muscle formation directly determines meat production and quality. The non-SMC condensin I complex subunit G (NCAPG) is strongly linked to the growth features of domestic animals because it is essential in controlling muscle growth and development. This study aims to elucidate the tissue expression level of the bovine *NCAPG* gene, and determine the key transcription factors for regulating the bovine *NCAPG* gene. In this study, we observed that the bovine *NCAPG* gene exhibited high expression levels in longissimus dorsi and spleen tissues. Subsequently, we cloned and characterized the promoter region of the bovine *NCAPG* gene, consisting of a 2039 bp sequence, through constructing the deletion fragment double-luciferase reporter vector and site-directed mutation-identifying core promoter region with its key transcription factor binding site. In addition, the key transcription factors of the core promoter sequence of the bovine *NCAPG* gene were analyzed and predicted using online software. Furthermore, by integrating overexpression experiments and the electrophoretic mobility shift assay (EMSA), we have shown that cAMP response element binding protein 1 (CREB1) and myogenic differentiation 1 (MYOD1) bind to the core promoter region (−598/+87), activating transcription activity in the bovine *NCAPG* gene. In conclusion, these findings shed important light on the regulatory network mechanism that underlies the expression of the *NCAPG* gene throughout the development of the muscles in beef cattle.

## 1. Introduction

Muscle growth and development are important factors that determine meat yield and quality, which are characteristics that have economic significance in intensive cattle ranching [1]. Myogenesis is a complex biological process that involves the proliferation and differentiation of myoblasts that fuse to form myotubes and mature muscle fibers [2,3,4]. Many regulatory factors, such as signaling molecules and transcription factors, precisely regulate this process [5]. Although a large number of studies have identified a series of key genes related to muscle growth and development through transcriptome sequencing and a genome-wide association study (*GWAS*), the regulatory mechanisms at the transcriptional level have been less studied.

A *GWAS* identified the *NCAPG* gene, which is linked to the growth and development of muscles. *NCAPG* is a subunit of condensin I located on the bovine sixth chromosome. It plays a crucial role in chromatin compression and chromosome segregation during mitosis and meiosis [6,7]. Some studies have shown that body length is often directly related to meat production [8,9]. The *NCAPG* gene has been found to be associated with body height [10,11,12] and demonstrated to play a role in muscle growth across different animal species [13,14,15]. Additionally, *NCAPG* gene expression potentially influences human height determination, as well as complex traits such as pig length, horse height, chicken carcass weight, and cattle growth [16,17,18,19,20]. In the meantime, on the basis of RNA-seq data and SNP analysis, the expression of the *NCAPG* gene exhibited significant differences between the fetal and adult longissimus dorsi muscle [21].

Additionally, *NCAPG* affects the formation of cardiomyocytes by modifying chromatin, which exacerbates heart disease [22]. In skeletal muscle myoblasts from Holstein dairy cows, the knockout of the *NCAPG* gene promotes cell apoptosis, extends mitosis, and impairs differentiation during the fetal bovine myoblast myogenic differentiation process [23]. Furthermore, interference in *NCAPG* gene expression results in impaired myoblast differentiation and myotube apoptosis [23]. Thus, it can be inferred that the regulatory process of *NCAPG* in organisms is highly intricate and diverse. In addition to the influence of the feeding conditions and nutrition level on meat yield and meat quality, transcriptional regulation of genetic factors also plays a key role in meat quality [24]. However, most current research on *NCAPG* has predominantly focused on human and rodent models, with limited studies conducted on ruminants. For example, in humans, *NCAPG* is regulated by chromobox protein homolog 3 (CBX3) transcription, which activates Wnt/β-catenin signaling and promotes colorectal cancer progression [25]. It was found that *NCAPG* positively regulates the activity of the E2F transcription factor 1 (E2F1) pathway in mice, promoting PARP1-E2F1 interaction to activate E2F1 target gene expression [26]. Therefore, additional compelling evidence is required to elucidate the role and mechanism of *NCAPG* in bovine myoblast development.

The binding of transcription factors to the promoter region’s core promoter sequence is a critical component of transcriptional regulation because it modifies gene transcription and, in turn, controls the expression of target genes. So far, there is no relevant research about the promoter region of bovine *NCAPG* in the literature. In order to clarify the significance of the promoter region in the transcriptional regulation of the *NCAPG* gene, our study constructed seven promoter deletion fragment vectors to identify the core promoter region. To further illustrate the transcription regulation mechanism of *NCAPG*, this study predicted and verified the related transcription factors MYOD1 and CREB1. Our research reveals an in vitro transcription regulation mechanism for *NCAPG*, expanding our knowledge of the regulation of *NCAPG* gene expression and offering a new insight for molecular breeding techniques and genetic improvement in beef cattle.

## 2. Results

### 2.1. The Isolation and Culture of Bovine Myoblasts

**Figure 1:**
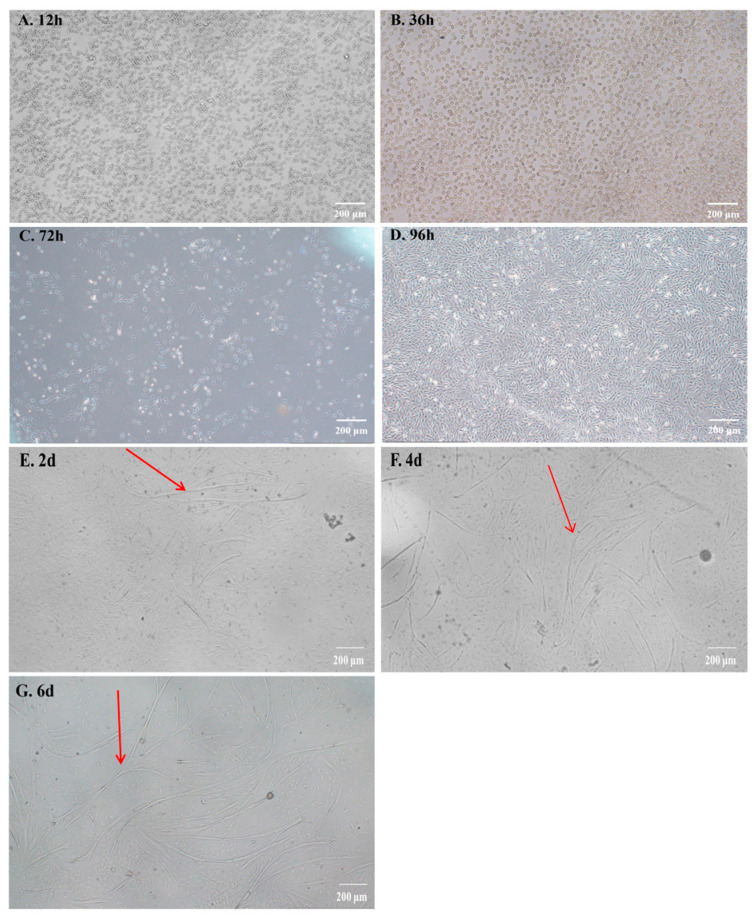
The proliferation and differentiation of bovine myoblasts. (**A**–**D**) Bovine myoblasts proliferated in different periods. (**E**–**G**) Bovine myoblasts induced differentiation in different periods. The red arrows indicate myotubes formed after induced differentiation; scale bar = 200 µm.

Following the method described by Zhen and Zhu for isolating and culturing muscle cells [27,28], we discovered that the majority of cells were digested by collagenase, adhered to the 25 cm^2^ cell culture bottle, and exhibited a pebble-like morphology within 12–36 h (Figure 1A,B). After a duration of 72 h, the cells underwent deformation, resulting in an elongated fused morphology, and displayed an increased rate of proliferation (Figure 1C). By 96 h, the cell density reached between 60% and 80%, with distinct alignment (Figure 1D). During induction differentiation, myoblasts progressively fused and differentiated into myotubes (Figure 1E–G).

### 2.2. Identification of Bovine Myoblasts

**Figure 2:**
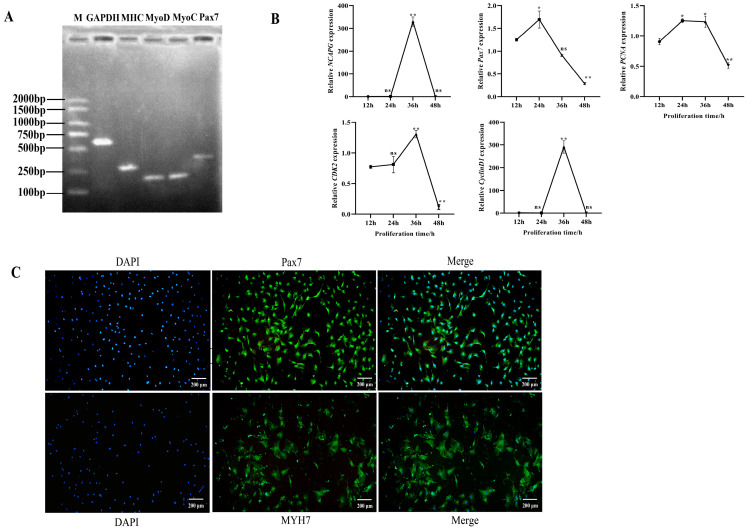
Identification of bovine myoblasts. (**A**) Expression of myoblast-related marker genes was determined via reverse transcription PCR. M: Marker; *GAPDH*: internal control. (**B**) The expression levels of *NCAPG*, *Pax7*, *PCNA*, *CDK2*, and *CyclinD1* genes at h 12, 24, 36, and 48 after bovine myoblast proliferation. The results are represented as the mean ± the SD based on three independent experiments. (**C**) Identification of bovine myoblasts based on Pax7 and MYH7 expression in growth medium for 48 h. The nucleus emits blue fluorescence, and the green spots indicate Pax7 and MYH7 fluorescence staining. Scale bar = 200 µm. * indicates significance at *p* < 0.05, and ** indicates significance at *p* < 0.01 compared to the control group; ns: not significant. Error bars represent the SD.

The regulatory factors Pax7, MHC, MYOD, and MYOC play crucial roles in myogenesis by influencing the fate determination of myoblast precursor cells and promoting myoblast proliferation. The RT-PCR results depicted the expression of marker genes *Pax7*, *MHC*, *MYOD*, and *MYOC* in isolated and purified bovine myoblasts (Figure 2A). Furthermore, distinct temporal intervals during cell proliferation revealed the mRNA expression of proliferation maker genes *Pax7*, *CDK2*, *PCNA*, and *CyclinD1*. The QRT-PCR results demonstrated that key genes associated with myogenesis exhibited an initial increase followed by a subsequent decline. The expression levels of *CDK2* and *CyclinD1* reached their peak at 36 h, while *Pax7* and *PCNA* peaked at 24 h. In the late proliferation, there was a significant decrease in the expression of these four maker genes (Figure 2B). Subsequently, immunofluorescence analysis was performed to identify the isolated cells based on the specific expression proteins Pax7 and MYH7. The results demonstrated the positive expression of Pax7 and MYH7 proteins in nearly all cells (Figure 2C). These findings collectively indicate that the isolated cells are highly pure myoblasts.

### 2.3. Structure Analysis of Bovine NCAPG and Phylogenetic Tree Construction

**Figure 3:**
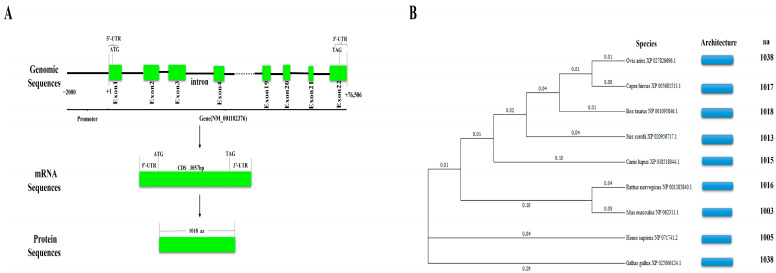
Sequence analysis and inferred phylogenetic tree of the *NCAPG* gene. (**A**) The detailed genomic, mRNA, and protein components containing the 5′/3′-untranslated region (5′/3′-UTR), and the CDS of bovine *NCAPG*. The figure omits the sequence from exon 5 to exon 18, as indicated by the dotted lines. (**B**) Phylogenetic tree of amino acids in the NCAPG protein of nine species, including *Bos taurus*. The left image shows the clustering of the NCAPG protein in different species, and the right shows the architecture and characteristics of the NCAPG protein amino acid length in corresponding species.

We characterized the bovine *NCAPG* gene structure and discovered that it is located on chromosome 6 and contains 22 exons and 21 introns. The length of the gene is approximately 76,506 bp. The NM_001102376.2 mRNA transcript of the gene is 3230 bp in length. We identified a coding sequence (CDS) of 3057 bp, which encoded 1018 amino acids (Figure 3A).

The phylogenetic tree reveals the relationship between the potential evolutionary processes of the NCAPG in different species. The bovine NCAPG amino acid sequence is highly similar to other mammals, especially among ruminant animals, for instance, sheep and goats. This result indicates that the NCAPG protein has a strong biological association among different species and the amino acid residual cardinal order is nearly equal (Figure 3B).

### 2.4. Schema of NCAPG Expression in Bovine Tissues and Organs

**Figure 4:**
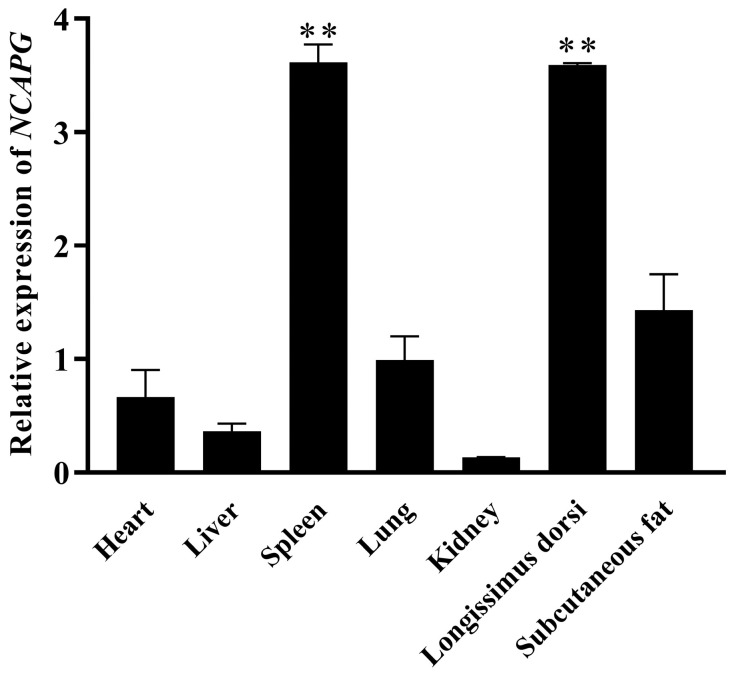
Gene expression of *NCAPG* in different tissues. The relative expression pattern of the *NCAPG* gene was detected using quantitative real-time PCR in a range of bovine tissues, and the lung was set as unit 1 to compare different tissues. The results are represented as the mean ± the SD based on three independent experiments. ** indicates significance at *p* < 0.01 compared to the control group. Error bars represent the SD.

In order to gain a comprehensive understanding of the transcriptional regulatory mechanisms of *NCAPG* in cattle, the tissue expression profiles of the bovine *NCAPG* gene were analyzed in different types of tissues and qPCR was performed using cDNA from tissues and organs, including the heart, liver, spleen, lung, kidney, longissimus dorsi, and subcutaneous fat. We found that *NCAPG* was significantly expressed in diverse organs and tissues, with the highest expression in the longissimus dorsi and spleen (Figure 4; *p* < 0.01). This result shows that the *NCAPG* gene might be strongly associated with muscle development.

### 2.5. Confirm of Bovine NCAPG Core Promoter Region

**Figure 5:**
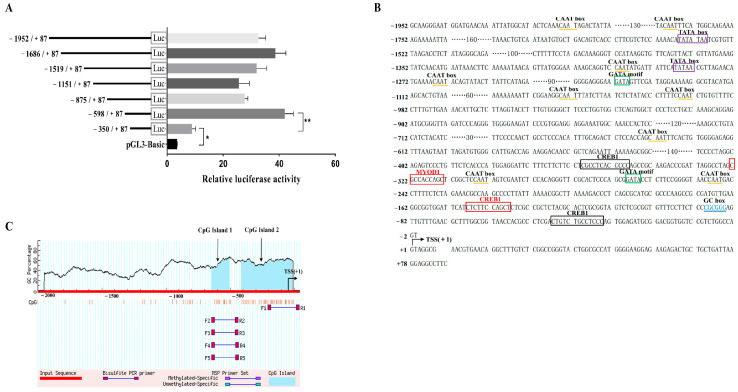
Construction of fragment deletion of the *NCAPG* gene promoter and the isolation and sequence analysis of the functional proximal minimal promoter of *NCAPG*. (**A**) Relative luciferase activity of different promoter fragments. These plasmids were transfected into C2C12. The results are expressed as the mean ± the SD in arbitrary units based on the firefly luciferase activity normalized against the Renilla luciferase activity for triplicate transfections. (**B**) The sequence of the 5′ regulatory region of bovine *NCAPG*. Arrows indicate the transcription initiation sites. The TF binding sites and regulatory elements are shown in boxes. (**C**) Schematic representation of the proximal promoter region (+87 to −1952 base pairs) of the bovine *NCAPG* gene to predict the regions with high GC content. Dashed lines indicate the GC percentage as represented by the *y*-axis, and the *x*-axis denotes the bp position in the 5′ untranslated region; vertical lines indicate the relative positions of CpG islands. Coordinates are given relative to the translational start site (shown as +1) * indicates significance at *p* < 0.05, and ** indicates significance at *p* < 0.01 compared to the control group. Error bars represent the SD.

To further explore the core region involved in transcriptional activity and potential key elements, we generated seven serial deletion reporter vectors from the 5′ end bovine *NCAPG* gene (Figure 5A). Subsequently, recombinant luciferase reporter plasmids were constructed by ligating *NheI* and *XhoI* restriction enzyme fragments into the pGL3-Basic vector (Figure 5B). These vector fragments were transfected into C2C12 cells to assess changes in luciferase activity (Figure 5C). Our results revealed a functional promoter located within the −1952/+87 region, as evidenced by an eight-fold increase in promoter activity compared to the pGL3-Basic vector. However, deletion from −1952 to −598 did not alter transcriptional activity. Notably, further deletions within the −598 to −350 bp and −350 to +87 regions resulted in reduced transcriptional activity. Therefore, our findings suggest that a core functional promoter exists in the upstream region 598 bp from the transcription initiation site.

In addition, the bioinformatics software was used to predict transcription factor binding sites from −598 to +87 bp (Figure 5D). Based on the prediction results and the relevant literature, four pivotal transcription factors, CREB1, SP1, MYOD1, and YY1, respectively, were selected to identify important positive regulatory elements. In the meantime, Methprimer’s online analysis discovered two CpG islands, and one is located in the *NCAPG* gene core promoter region (−598 to +87) (Figure 5E). Moreover, the identified transcription factor binding sites were found within the CpG island.

### 2.6. Validation of the TF Functions

**Figure 6:**
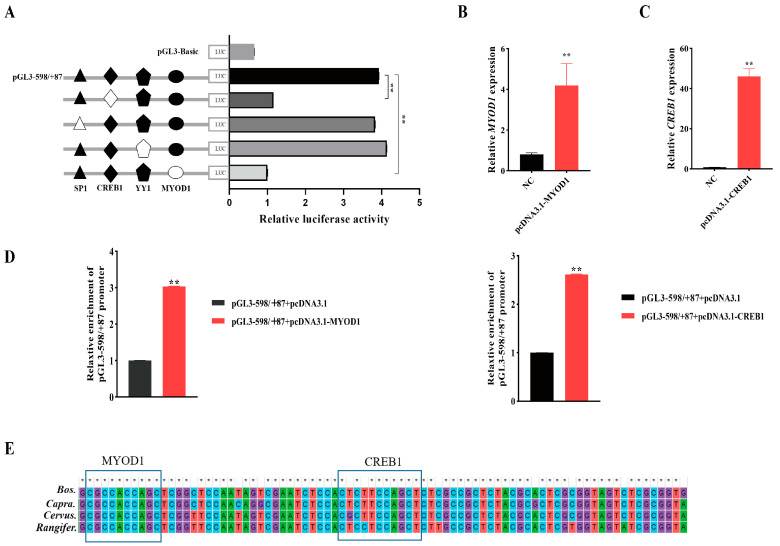
Analysis of transcriptional activity for the corresponding transcription factor in bovine myoblast cells. (**A**) Site-directed mutagenesis for MYOD1 and CREB1 sites was carried out in the construct pGL−598/+87. The correspondence constructs were transiently transfected into myoblasts, and the pGL−598/+87 construct was used as a negative control (NC). White and black filled shaped in sequence depictions represent wild and mutant types, respectively. (**B**,**C**) Overexpression efficiency of pcDNA3.1-*MYOD1* and pcDNA3.1-*CREB1*. The pcDNA3.1(+) empty vector was used as a negative control (NC). (**D**) Luciferase reporter assays after MYOD1 and CREB1 overexpression via co-transfection with pGL−598/+87 in bovine myoblasts. The pGL3−598/+87 and pcDNA3.1(+) empty-vector co-transfection groups were used as a negative control (NC). (**E**) Multiple sequence alignment of the MYOD1 and CREB1 transcription factor binding sites in the promoter region of the *NCAPG* gene in different species. The box depicts MYOD1 and CREB1 consensus binding sites. * indicates significance at *p* < 0.05, and ** indicates significance at *p* < 0.01 compared to the control group. Error bars represent the SD.

To elucidate the roles of these transcription factors (TFs) in the regulation of *NCAPG,* a series of site-directed mutagenesis plasmids containing TFs were constructed within the pGL−598/+87 vector and transfected into bovine myoblasts (Figure 6A). The results demonstrated that mutations in the CREB1 and MYOD1 sites in the pGL−598/+87 vector significantly attenuated transcriptional activity (*p* < 0.05).

To further validate the involvement of CREB1 and MYOD1 in the regulation of bovine *NCAPG* promoter activity, a co-transfection approach was employed to introduce an overexpression vector containing both CREB1 and MYOD1 into bovine myoblasts, along with pGL−598/+87. Initially, we assessed the transfection efficiency by examining the overexpression levels of CREB1 and MYOD1 in bovine myoblasts (Figure 6B,C). The results demonstrated a significant upregulation of these transcription factors. Subsequently, our findings also revealed that the overexpression of CREB1 and MYOD1 substantially augmented the activity of the *NCAPG* core promoter (Figure 6D).

Furthermore, we analyzed the conservation of CREB1 and MYOD1 transcription factors across various species, including cattle, goats, cervus, and rangifer. These findings provide compelling evidence for the pivotal role of the CREB1 and MYOD1 elements in the regulation process of *NCAPG* transcription (Figure 6E).

### 2.7. DNA–Protein Interactions through Electrophoretic Mobility Shift Assays

**Figure 7:**
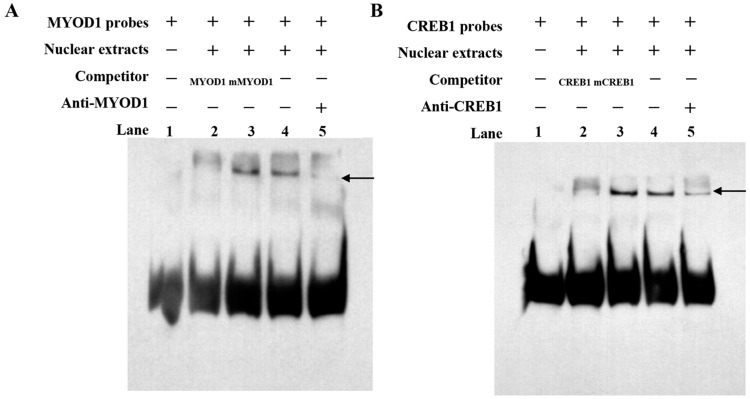
Identification of TF binding to the core promoter of *NCAPG* using EMSA. (**A**) EMSA tests confirmed the binding of the transcription factor MYOD1 to the *NCAPG* promoter in vitro. (**B**) EMSA tests confirmed the binding of the transcription factor CREB1 to the *NCAPG* promoter in vitro. Nuclear protein extracts were incubated with a 5′ biotin-labeled probe containing the MYOD1 and CREB1 binding site in the presence or absence of a competitor (lane 4), mutation probe (lane 3), and unlabeled probe (lane 2). The super-shift assay was conducted using 10 μg of Anti-MYOD1 and Anti-CREB1 antibodies (lane 5). Arrows indicate DNA–nuclear-protein complexes.

EMSA was performed to determine CREB1 and MYOD1 binding sites on the *NCAPG* promoter. As depicted in the figure, DNA–protein complexes were formed when the 5′ bio-labeled CREB1 and MYOD1 probes were bound to the nuclear proteins of bovine myoblasts (lane 4, Figure 7A,B). After incubation with specific non-biotin units and probes, the nuclear proteins showed the disappearance of the protein–DNA complex (lane 2, Figure 7A,B). In contrast, the protein–DNA complex was minimally affected by the addition of mutational probes (lane 3, Figure 7A,B). Following incubation with anti-CREB1 and anti-MYOD1 antibodies, the binding of the antibodies to CREB1 and MYOD1 results in weak binding of the probe to transcription factors, weak migration bands, and an over-shift in the complex (lane 5, Figure 7A,B). These results indicate the specific binding of CREB1 and MYOD1 transcription factors to the *NCAPG* gene core promoter sequence in vitro, validating their role in the regulation of the *NCAPG* gene.

### 2.8. Effect of NCAPG on Genes Involved in Myogenesis in Bovine Myoblasts

**Figure 8:**
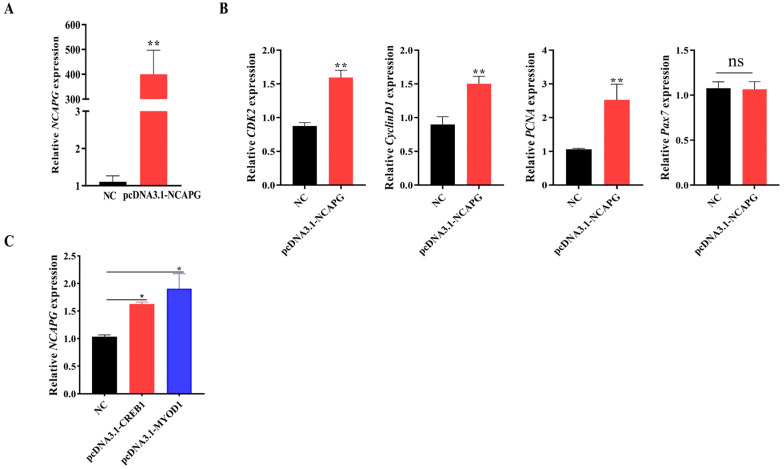
*NCAPG* regulated the relative expression of myogenesis marker genes. (**A**) Overexpression efficiency of pcDNA3.1-*NCAPG*. The pcDNA3.1(+) empty vector was used as negative control (NC). (**B**) The relative expression levels of marker genes in bovine myoblasts. (**C**) The *NCAPG* mRNA relative expression after transfection with different TF overexpression vectors. The pcDNA3.1(+) empty vector was used as a negative control (NC). * indicates significance at *p* < 0.05, and ** indicates significance at *p* < 0.01 compared to the control group. ns: not significant. Error bars represent the SD.

In order to investigate the genetic and molecular regulatory mechanisms of *NCAPG* in the process of myogenesis in myoblasts, the mRNA expression levels of pertinent marker genes associated with myogenesis and bovine myoblast development were examined. *NCAPG* overexpression (pcDNA3.1-*NCAPG*) led to a significant increase in the *NCAPG* mRNA expression level (Figure 8A). The overexpression of *NCAPG* in bovine myoblasts has been shown to be effective and successful. Furthermore, pcDNA3.1-*NCAPG* myoblasts exhibited higher mRNA expression levels of *CDK2*, *CyclinD1*, and *PCNA* compared to the pcDNA3.1(+) empty vector (NC) (Figure 8B). However, no significant alteration was observed in *Pax7* gene expression (Figure 8B). The expression level of *NCAPG* increased significantly with the overexpression of *CREB1* and *MYOD1* (Figure 8C). These findings indicated that *NCAPG* overexpression can activate myogenesis genes in myoblasts.

## 3. Discussion

Muscle is a complicated tissue that develops at a highly periodic rate and directly affects the yield of meat carcasses. Transcription factors are fundamental transcription elements that govern the expression of target genes and drive cell differentiation, thereby impacting various biological traits [29]. During different stages of myogenesis, the bovine NCAPG has been observed to localize in both the cytoplasm and nucleus, playing a significant role in regulating the expression of genes associated with myogenesis [23]. Liu’s study revealed a substantial level of *NCAPG* gene expression in fetal and adult muscles in Qinchuan cattle [21]. *NCAPG* is essential in mammalian cell division atrophy and is closely linked to domestic animals’ physique [30]. Studies in recent years have discovered that the *NCAPG* gene not only regulates the growth and differentiation of cancer cells but also influences muscular development in animals [31]. In this work, we found that the longissimus dorsi and spleen express the *NCAPG* gene at a high level and that proliferation marker genes are significantly expressed at different stages of proliferation. The composition and characteristics of muscle fibers in muscle tissue are influenced by a multitude of factors, including external environmental conditions and individual genetics [32]. The longissimus dorsi (LD) muscle is a crucial component of animal muscle anatomy, closely associated with the growth and development of skeletal muscles and the intramuscular fat content in animals [33]. Studies have demonstrated that bovine LD muscles contain a high proportion of fast-twitch (Type II) fibers, which have larger diameters than slow-twitch (Type I) fibers, favoring muscle hypertrophy [34]. Hepatocyte growth factor (HGF) is present in skeletal muscles and promotes skeletal muscle regeneration by activating quiescent satellite cells. Research indicates that in rats, HGF expression levels in the spleen significantly increase following muscle injury, suggesting that spleen-derived HGF may participate in the activation and proliferation of skeletal muscle satellite cells during muscle regeneration [35]. Therefore, the analysis of amino acid sequences indicates a remarkable conservation of its protein across different species’ evolution. In summary, the *NCAPG* gene plays a crucial role in individual growth and development.

According to recent research, *NCAPG* is situated within quantitative trait loci (QTL) that control important traits, including body frame size and carcass weight [36]. These findings suggest a significant involvement of *NCAPG* in the phenotype of cattle. For our research, we obtained the promoter sequence of the bovine *NCAPG* gene and identified two CpG islands within a 2 kb region of its promoter using online software analysis. Notably, one of these CpG islands is located in the core promoter region (399bp). Generally, transcription commences at core promoters situated around the 5′ initiation site of genes, and the core promoter recruits transcription factors through polymerase II, which determines the precise location and direction of transcription initiation [37]. DNA methylation, an essential epigenetic modification, is indispensable for mammalian development and plays a pivotal role in the recognition and maintenance of cellular identity [38]. Generally, methylation levels typically impact mRNA expression [39]. Previous research has demonstrated that DNA methylation of the bovine *Six1* gene promoter significantly influences promoter activity and exhibits a negative correlation with *Six1* gene expression [40]. In studies on the *Six1* gene in pigs, the methylation of the core promoter region showed tissue-specific expression patterns, with low methylation levels observed in skeletal and longissimus dorsi muscles but higher levels in the masseter muscle [41]. Based on these findings, we hypothesize that DNA methylation at CpG sites within the core promoter of the *NCAPG* gene would regulate gene expression [42], thereby affecting the processes of formation and development of bovine muscle, offering important new angles for future research on the epigenetic modification of the *NCAPG* gene.

The expression levels of *CDK2*, *PCNA*, and *Cyclin D1*, genes involved in the proliferation of bovine myoblasts, were found to be strongly impacted by the overexpression of *NCAPG*. Previous studies have reported that the increased expression of *CDK2*, *PCNA*, and *CyclinD1* promotes the proliferation of bovine myoblasts [43,44]. Silencing the *FOXO1* gene can promote the expression of *PCNA* and *CDK2* in bovine myoblasts [45]. In short, previous research and our own findings suggest that *NCAPG* may enhances bovine myoblasts’ proliferation by regulating the expression of key proliferation marker genes and have a positive effect on muscle production.

The core region of the *NCAPG* gene was successfully identified through the approach using the sequential elimination of luciferase activity. Utilizing online prediction software, we observed that the nucleotide sites within the potential transcription factor binding sites MYOD1 and CREB1 were highly conserved among herbivores. Subsequently, site-directed mutagenesis was performed on the core promoter sequences of MYOD1 and CREB1, the transcription factors, resulting in a significant reduction in luciferase activity. Furthermore, EMSA results demonstrated that MYOD1 and CREB1 transcription factors bind to the core promoter region of the *NCAPG* gene.

MYOD1 belongs to the MYOD protein family, which is essential for myogenesis and muscle differentiation. It is also thought to be a possible candidate gene for characteristics related to meat production in livestock [46,47,48,49]. In a relevant study conducted on Guanling cattle, it has been confirmed that MYOD1 is closely associated with muscle formation and differentiation [50]. Furthermore, MYOD1 regulates muscle growth and development by recruiting transcription factors such as c-Jun, Jdp2, Meis, and Runx1, thereby enhancing the expression of genes related to muscular characteristics [51]. Single-nucleotide polymorphism (SNP) markers identified in porcine MYOD1 may influence the expression level of this gene and have significant effects on muscle fiber characteristics, lean meat yield, and meat quality [52]. Sun et al. reported that *MEF2A* positively modulates bovine myoblast proliferation by controlling the expression of *MYOD1*, an essential growth-related gene [53]. The impact of MYOD1 expression on beef tenderness may be attributed to its role as a transcriptional protein for muscle-specific genes, such as the serum response factor (SRF) gene. SRF plays a crucial role in regulating muscle tissue development, composition, growth, and maturation [54]. According to recent studies, the bovine *MYOD1* gene functions as a transcription factor that is crucial for regulating the transcription of the *LAST1* gene [46].

Activating transcription factor-1 (ATF-1), CREB, and CRE modulator (CREM) are members of the leucine zipper family, which also includes cyclic AMP response element binding protein 1 [55,56]. The expression of *CREB* is regulated by various intracellular factors, including cAMP, intracellular calcium ions, and growth factors, and it exhibits high abundance in the liver and skeletal muscles [57]. Feng’s findings suggest that CREB1 may play a significant role in cell proliferation, apoptosis, myogenic differentiation, and skeletal muscle repair pathways [58]. Numerous studies have demonstrated that CREB1 exerts control over the growth and invasion of cancer cells, nerve cells, vascular endothelial cells, and immunized T cells [59,60]. Previous studies have demonstrated that CREB1 facilitates the myogenic differentiation of mouse C2C12 cells by regulating *NRMT1* transcription [61]. The presence or absence of a 26 bp insertion in the *CREB1* gene is significantly associated with growth traits in sheep, such as body length and height [62]. The cooperative action of *CREB1*, *MEF2C*, and *MYOD* forms a triple complex that drives the expression of *Tceal7*, a striated muscle-specific gene, during skeletal muscle regeneration [63]. In mice suffering from acute muscle damage, phosphorylated *CREB1* promotes myoblast proliferation and improves muscle regeneration [64].

**Figure 9:**
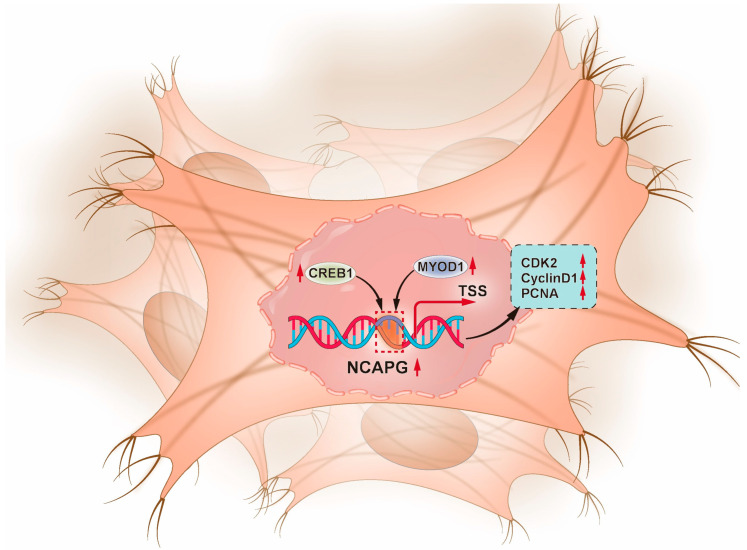
A model summary of *NCAPG* regulation by MYOD1 and CREB1 transcription factors. In short, MYOD1 and CREB1 increased the *NCAPG* promoter activity, and the overexpression of *NCAPG* enhanced the expression of marker genes of *CDK2*, *CyclinD1*, and *PCNA*.

In summary, the existing literature has consistently highlighted the regulatory roles played by MYOD1 and CREB1 transcription factors in these crucial processes, particularly myogenesis. Thus, this study suggests that the *NCAPG* gene, whose key transcriptional regulatory elements are MYOD1 and CREB1, critically regulates bovine myogenesis. In this study, there are some limitations. We have focused on screening and identifying important transcription factors potentially involved in the regulation of bovine *NCAPG* gene transcription. However, the detailed regulatory mechanisms by which these key transcription factors influence *NCAPG* gene activity remain inadequately understood. Therefore, we plan to perform functional verification at the cellular level in vitro in the future. This approach aimed to enhance our understanding of the molecular regulatory mechanism of *NCAPG* gene in bovine muscle development. A proposed model of the transcriptional regulatory system of the bovine *NCAPG* gene is provided in Figure 9.

## 4. Materials and Methods

### 4.1. Sample Collection and Preparation

The tissue samples utilized in this study, including the heart, liver, spleen, lung, kidney, longissimus dorsi, and subcutaneous fat of three 1.5-year-old cattle, were collected from the livestock farm of Gansu Agricultural University (Lanzhou, China). Subsequently, these samples were promptly immersed in liquid nitrogen and subsequently stored at −80 °C. Myoblasts were isolated from 7-day-old cattle.

### 4.2. Isolation, Identification, and Culture Bovine Myoblasts

The separation of bovine myoblasts was achieved using the collagenase digestion method. Briefly, muscle blocks were rinsed by rinsing m with sterile phosphate-buffered saline (PBS, G4202, Servicebio, Wuhan, China) (PH 7.3, stored at 4 °C). Sterilized scissors and forceps were then utilized to remove connective tissue, such as the sarcolemma, and cut it into tissue chyme measuring less than 1 cm^3^. Subsequently, the tissue was digested with 0.25% collagenase II (C8150, Solarbio, Beijing, China) (stored at −20 °C) for release of cells. Finally, the cell suspension was filtered through a cell sieve (BS-70-XBS, Biosharp, Beijing, China). After collecting the cell clumps, they should be washed three times with PBS, centrifuged at 1000 rpm/min, and then resuspended in the primary myoblast growth medium containing 80% DMEM/F12 (C11330500BT, Hyclone, New York, NY, USA) (stored at 4 °C), 20% fetal bovine serum (A6904, Invigentech, Carlsbad, CA, USA) (stored at −20 °C). Subsequently, the cells should be placed in a 5% CO_2_ incubator (CCL-170T-8, ESCO, Shanghai, China) set at 37 °C. Following the method described by Zhen [28], myoblast differentiation was induced, and morphological changes in the cells were observed.

To obtain relatively pure myoblasts, the floating cells can be serially transferred to a new culture dish based on their differential attachment times, effectively eliminating fibroblasts, and other impurities. Subsequently, we assessed the purity of myoblast cells by performing reverse-transcription–PCR immunofluorescence staining using specific anti-Pax7 and anti-MYH7 antibodies (TA7584S and TD12122S, Abmart, Shanghai, China) (stored at −20 °C), and evaluation of marker gene expression. Briefly, the myoblasts were cultured in 24-well cell culture dishes (CCP-24H, Servicebio, Wuhan, China) until reaching a cell density of 60–70%. The medium was then removed, and each well was fixed with 200 μL 4% paraformaldehyde (G1101, Servicebio, Wuhan, China) (store at 4 °C) for 30 min. Following fixation, the permeabilization was performed using 0.5% Triton X-100 (G3068, Servicebio, Wuhan, China) (stored at room temperature) for 20 min, followed by blocking with 5% BSA (GC305010, Servicebio, Wuhan, China) (stored at 4 °C) for an additional 30 min. For immunofluorescence analysis, the cells were incubated overnight at 4 °C with primary antibody diluted in 5% BSA (1:250). After being washed with PBS, the cells were incubated with a secondary antibody, Goat Anti-Rabbit IgG (H&L) FITC (M212315S, Abmart, Shanghai, China) (stored at −20 °C), diluted in 5% BSA at a ratio of 1:500. The samples were stored at room temperature in a light-protected environment for 2 h. DAPI (G1012, Servicebio, Wuhan, China) (stored at 4 °C) was used at the final concentration of 1 μg/mL to stain the nuclei under dark conditions at 37 °C for 10 min. Finally, immunofluorescence images were captured using the Olympus IX73 microscope (IX73, Olympus, Tokyo, Japan).

### 4.3. DNA, RNA Isolation, and mRNA Expression Analysis

The genomic DNA from longissimus dorsi was extracted using the traditional phenol/chloroform isolation method, as described by Ye et al. [65]. Total RNA from tissues and cells was extracted using TRIzol reagent (R411, Vazyme, Nanjing, China) (stored at 4 °C). The concentration and purity of RNA were determined using the NanoDrop 8000 spectrophotometer (ND8000-GL, NanoDrop Technologies, Wilmington, NC, USA). Following the guidelines provided in the TransScript One-Step gDNA Removal and cDNA Synthesis SuperMix instruction manual (AT311, Transgen, Beijing, China) (stored at −20 °C), cDNA synthesis was performed.

In addition, qRT-PCR was conducted using the PerfectStart Green qPCR SuperMix (AQ601, Transgen, Beijing, China) (stored at −20 °C) with an Applied Biosystems QuantStudio 6 Flex Real-time PCR System (4485692, Thermo Fisher Scientific, Waltham, MA, USA). For each reaction, 2 μL of cDNA (equivalent to 400 ng of diluted cDNA for 20 μL quantitative PCR) was added and amplified for 40 cycles under the following procedure: denaturation for 15 s at 95 °C and annealing for 30 s at 60 °C. Then, the dissolution curve was amplified via pre-denaturation for 10 s at 95 °C, denaturation for 1 min at 60 °C, and annealing for 15 s at 95 °C. The experiments were performed in triplicate, and data analysis was carried out by employing the 2^−ΔΔCt^ method. To normalize the mRNA expression level, *GAPDH* served as an internal control [66]. A comprehensive list of all the primers examined in this study can be found in Table S1.

### 4.4. Promoter Cloning and Sequence Structure Analysis

To identify the bovine *NCAPG* gene’s promoter sequences, we used the NCBI reference genome and selected the bovine option (version number: ARS-UCD2.0), https://www.ncbi.nlm.nih.gov/ (accessed: 20 July 2023). By entering the *NCAPG* gene entry number (NM_001102376), we searched for the 5 ‘UTR sequence of the *NCAPG* gene, considering the first exon sequence start site as +1. We then selected sequences from upstream −1952 bp to downstream +87 bp, approximately 2039 bp, as the candidate promoter full-length promoter sequences. We identified a putative promoter region for the bovine *NCAPG* gene (NC_037333.1, from 37330072 to 37332111). The promoter region was amplified using specific primers (*NCAPG*-PF/PR) and genomic DNA as the template. Subsequently, we obtained the promoter sequence through agarose gel electrophoresis and sequencing. Furthermore, various bioinformatics software tools were employed to investigate the structure, amino acid composition, and homology of the bovine *NCAPG* gene. Consequently, the potential binding sites of transcription factors within *NCAPG* promoter were analyzed using AliBaba2.1, http://gene-regulation.com/pub/programs/alibaba2/index.html (accessed: 23 July 2023), and AnimalTFDB, http://bioinfo.life.hust.edu.cn/AnimalTFDB4/#/ (accessed: 24 July 2023) [67]. CpG islands were predicted using MethPrimer, https://www.urogene.org/methprimer/ (accessed: 24 July 2023), and the phylogenetic tree was built by comparing amino acid sequences from nine different species with MEGA7.0 software [68].

### 4.5. Construction of Promoter Luciferase Reporter Vectors

The 2039 bp bovine NCAPG promoter fragment was purified, digested with restricted enzymes, and then ligated to pGL3-Basic vector using T4 DNA Ligase (FL101, Transgen, Beijing, China) (stored at −20 °C) to obtain the luciferase reporter vector. The monoclonal selection was performed using Trans5α Chemically Competent Cell (CD201, Transgen, Beijing, China) (stored at −20 °C), and recombinant fragments were previously digested with NheI and XhoI restriction enzymes (JN301 and JX201, Transgen, Beijing, China) (stored at −20 °C). The resulting plasmid was named NCAPG-F1 (−1952/+87). Based on transcription factor binding sites of the bovine NCAPG gene, seven different fragments were designed via amplification, with NCAPG-F1 as a template. These fragments included the deletion of portions of the bovine NCAPG gene promoter and were named as follows: NCAPG-F2 (−1686/+87), NCAPG-F3 (−1519/+87), NCAPG-F4 (−1151/+87), NCAPG-F5 (−875/+87), NCAPG-F6 (−598/+87), and NCAPG-F7 (−350/+87).

### 4.6. Cell Culture, Transfection, and Relative Activity of Dual-Luciferase Assay

C2C12 cells (TCM-C720, Haixing Biosciences, Suzhou, China), kindly provided by Suzhou Haixing Biosciences Co., Ltd., were cultured in Dulbecco’s Modified Eagle Medium with high glucose (DMEM-H) (C11995500BT, GIBCO, Grand Island, NY, USA) (stored at 4 °C), supplemented with 10% fetal bovine serum (A6904, Invitrogen, Carlsbad, CA, USA) (stored at −20 °C) and 1% penicillin/streptomycin (P/S) (15140, GIBCO, Grand Island, NY, USA) (stored at −20 °C). The cells were incubated at 37 °C under standard humidity conditions and a CO_2_ concentration of 5%. Upon reaching a cell density of 70–80%, the culture was passaged.

Subsequently, C2C12 cells were seeded in 24-well plates at a density of 1 × 10^6^ per well in a growth medium. Co-transfections of 800 ng of seven recombinant plasmid vectors (*NCAPG*-F1 to *NCAPG*-F7) and 10 ng pRL-TK plasmid were performed using the INVI DNA and RNA Transfection Reagent^TM^ (IV1216150, Invigentech, Carlsbad, CA, USA) (stored at −20 °C). The pRL-TK vector served as an internal reference for result calibration, while the pGL3-Basic vector was used as a negative control. After transfection for 48 h, cell lysates were collected using a cell Lysis buffer (FR201, Transgen, Beijing, China) (store at −20 °C), and the dual-luciferase activity was measured with a microplate reader (VL0000D0, Thermo Scientific, Waltham, MA, USA). Finally, the ratio of Firefly and Renilla luciferase yielded the relative transcription activity of unidirectional deletion series within the bovine *NCAPG* gene promoter.

### 4.7. Site-Directed Mutagenesis

The potential binding sites for transcription factors (TFs) on the promoter region of *NCAPG* were analyzed using AliBaba2.1 and AnimalTFDB databases. Putative TF binding sites for SP1, CREB1, MYOD1, and YY1 were mutated to investigate their role in myogenesis using specific primers (Table S1) using the Quick Change Site-Directed Mutagenesis Kit (200518, Stratagene, La Jolla, CA, USA) (stored at −20 °C). Briefly, following the manufacturer’s instructions, the PCR reaction mixture was prepared by adding 5 µL of 10× reaction buffer, 5 µL (25 ng) plasmid, 1.25 µL (125 ng) forward primer, 1.25 µL (125 ng) reverse primer, 1 µL dNTP mix, 1.5 µL QuickSolution reagent, 34 µL ddH_2_O, and 1 µL QuikChange^®^ Lightning Enzyme in sequence. The PCR products were then purified, recovered and transformed into receptor cells, and the resultant products were cultured for plasmid extraction. The transfection procedure was based on reference 4.6. The pRL-TK served as the internal reference plasmid, and pGL3-Basic was the negative control.

### 4.8. MYOD1, CREB1, and NCAPG Overexpression

To target the transcription factors (TFs) myogenic differentiation 1 (MYOD1), cAMP-responsive element binding protein 1 (CREB1), and target gene *NCAPG,* we amplified their sequences, using bovine myoblast cDNA as a template. The eukaryotic expression vector pcDNA3.1(+) and the synthetic *NCAPG*, *MYOD1*, and *CREB1* sequences were digested by three groups of endonuclease enzymes, namely *NotI* (JN401, Transgen, Beijing, China) and *XbaI* (JX101, Transgen, Beijing, China), *BamHI* (JB101, Transgen, Beijing, China) and *XhoI* (JX201, Transgen, Beijing, China), and *BamHI* and *XbaI*, respectively. After purification, the purified fragments were ligated with T4DNA ligase (FL101, Transgen, Beijing, China) at a ratio of 3:1 at 16 °C overnight. The ligation product was subsequently transformed into Trans5α Chemically Competent Cell (CD201, Transgen, Beijing, China). The transformed cells were planted on Luria–Bertani agar (A8190, Solarbio, Beijing, China) (stored at room temperature) containing ampicillin (A6920, Solarbio, Beijing, China) (stored at 4 °C). The positive clones were identified via PCR (pcDNA3.1(+) consensus primer F (5′- CATGAGAACCCACTGCTTAC-3′), R (5′- TAGAAGGCACAGTCGAGG-3′)). DNA of positive clones were mixed and amplified for 32 cycles under the following procedure: denaturation for 30 s at 94 °C, annealing for 45 s at 58 °C, and extension 50 s at 72 °C. Then, the amplified products were identified via restriction enzyme digestion and agarose gel analysis. Finally, the recombinant plasmid was prepared with the Endofree Mini Plasmid Kit II (DP118, TIANGEN, Beijing, China) (stored at 4 °C), namely, pcDNA3.1-*MYOD1*, pcDNA3.1-*CREB1,* and pcDNA3.1-*NCAPG*. When the density of myoblasts in the 6-well plate (CCP-6, Servicebio, Wuhan, China) reached 80%, using INVI DNA and RNA Transfection Reagent^TM^ (IV1216150, Invigentech, Carlsbad, CA, USA) following manufacturer’s instructions, the recombinant plasmid transfection of bovine myoblasts was carried out in a 6-well plate. Subsequently, 7.5 μg/well recombinant plasmid and pcDNA3.1(+) empty vector were transfected separately into bovine myoblasts to investigate their regulatory effects on *NCAPG* transcription activity. The pcDNA3.1(+) empty vector was used as negative control (NC). Three independent replicate experiments were performed for each group.

### 4.9. Electrophoretic Mobility Shift Assays

Bovine myoblasts in the logarithmic growth phase were inoculated into a T25 culture flask (CCF-T25H, Servicebio, Wuhan, China). Bovine cytoplasmic protein was extracted using the Nuclear Protein Extraction Kit (R0050, Solarbio, Beijing, China) (stored at −80 °C), and all DNA probes (Table S1) were synthesized (Invitrogen) and biotin-labeled at the 5′ end. Briefly, the binding reaction mixture containing 2 μL of 5× binding buffer, 10 μg of nuclear extracts, and 1 μL poly (dI-dC) in a 20 μL volume for 15 min on ice. Then, 200 fmol biotin DNA was incubated at room temperature for 20 min. For the competition assay, unlabeled probes or mutated probes were added to the reaction mixture 15 min before adding the labeled probes. For the super-shift assay, antibodies (10 μg) were included in the reaction and pre-incubated at a low temperature for 30 min before adding the labeled probes: anti-MYOD1 (TA7733S, Abmart, Shanghai, China) and anti-CREB1 (PS02122, Abmart, Shanghai, China). Subsequently, 6% non-denaturing polyacrylamide gel electrophoresis, using a duration polyacrylamide of one h with 0.5·tris-borate EDTA buffer, was employed to isolate the DNA–protein complex. The images were captured using the HQ-350XT+ system (HQ-350XT, Hu Qiu, Suzhou, China).

### 4.10. Statistical Analysis

The data are presented as mean ± SD from three independent experiments, and statistical analysis was performed using SPSS 22.0. Multiple-group comparisons were conducted using one-way ANOVA, while two-group comparisons were analyzed using a two-tailed Student’s *t*-test.

## 5. Conclusions

Bovine myoblasts were successfully separated and identified, and the results show that *NCAPG* expression was significantly upregulated in the muscle tissue of bovine. The core promoter region of the bovine *NCAPG* gene was precisely mapped within the range of −598 to +87 bp and was found to be regulated by MYOD1 and CREB1 TFs. Moreover, we verified that *NCAPG* overexpression may affect myoblast proliferation. These findings not only contribute to a deeper comprehension of the transcriptional regulation of the bovine *NCAPG* gene but also offer innovative molecular breeding strategies aimed at enhancing beef cattle yield and quality in the future.

## Data Availability

The dataset supporting the conclusions of this article is available via email to the corresponding author.

## Supplementary Materials

The following supporting information can be downloaded at: https://www.mdpi.com/article/10.3390/ijms25052506/s1.

## References

[1] Picard B., Gagaoua M. (2020). Muscle Fiber Properties in Cattle and Their Relationships with Meat Qualities: An Overview. J. Agric. Food Chem..

[2] Dayton W.R., White M.E. (2008). Cellular and molecular regulation of muscle growth and development in meat animals. J. Anim. Sci..

[3] Asfour H.A., Allouh M.Z., Said R.S. (2018). Myogenic regulatory factors: The orchestrators of myogenesis after 30 years of discovery. Exp. Biol. Med..

[4] Fu Y., Shang P., Zhang B., Tian X., Nie R., Zhang R., Zhang H. (2021). Function of the Porcine TRPC1 Gene in Myogenesis and Muscle Growth. Cells.

[5] Mohammadabadi M., Bordbar F., Jensen J., Du M., Guo W. (2021). Key Genes Regulating Skeletal Muscle Development and Growth in Farm Animals. Animals.

[6] Kimura K., Cuvier O., Hirano T. (2001). Chromosome condensation by a human condensin complex in Xenopus egg extracts. J. Biol. Chem..

[7] Zhang W., Li J., Guo Y., Zhang L., Xu L., Gao X., Zhu B., Gao H., Ni H., Chen Y. (2016). Multi-strategy genome-wide association studies identify the DCAF16-NCAPG region as a susceptibility locus for average daily gain in cattle. Sci. Rep..

[8] Mikawa S., Morozumi T., Shimanuki S., Hayashi T., Uenishi H., Domukai M., Okumura N., Awata T. (2007). Fine mapping of a swine quantitative trait locus for number of vertebrae and analysis of an orphan nuclear receptor, germ cell nuclear factor (NR6A1). Genome Res..

[9] Takasuga A., Sato K., Nakamura R., Saito Y., Sasaki S., Tsuji T., Suzuki A., Kobayashi H., Matsuhashi T., Setoguchi K. (2015). Non-synonymous FGD3 Variant as Positional Candidate for Disproportional Tall Stature Accounting for a Carcass Weight QTL (CW-3) and Skeletal Dysplasia in Japanese Black Cattle. PLoS Genet..

[10] Gudbjartsson D.F., Walters G.B., Thorleifsson G., Stefansson H., Halldorsson B.V., Zusmanovich P., Sulem P., Thorlacius S., Gylfason A., Steinberg S. (2008). Many sequence variants affecting diversity of adult human height. Nat. Genet..

[11] Lettre G., Jackson A.U., Gieger C., Schumacher F.R., Berndt S.I., Sanna S., Eyheramendy S., Voight B.F., Butler J.L., Guiducci C. (2008). Identification of ten loci associated with height highlights new biological pathways in human growth. Nat. Genet..

[12] Weedon M.N., Lango H., Lindgren C.M., Wallace C., Evans D.M., Mangino M., Freathy R.M., Perry J.R., Stevens S., Hall A.S. (2008). Genome-wide association analysis identifies 20 loci that influence adult height. Nat. Genet..

[13] Al-Mamun H.A., Kwan P., Clark S.A., Ferdosi M.H., Tellam R., Gondro C. (2015). Genome-wide association study of body weight in Australian Merino sheep reveals an orthologous region on OAR6 to human and bovine genomic regions affecting height and weight. Genet. Sel. Evol..

[14] Tetens J., Widmann P., Kuhn C., Thaller G. (2013). A genome-wide association study indicates LCORL/NCAPG as a candidate locus for withers height in German Warmblood horses. Anim. Genet..

[15] Signer-Hasler H., Flury C., Haase B., Burger D., Simianer H., Leeb T., Rieder S. (2012). A genome-wide association study reveals loci influencing height and other conformation traits in horses. PLoS ONE.

[16] Carty C.L., Johnson N.A., Hutter C.M., Reiner A.P., Peters U., Tang H., Kooperberg C. (2012). Genome-wide association study of body height in African Americans: The Women’s Health Initiative SNP Health Association Resource (SHARe). Hum. Mol. Genet..

[17] Eberlein A., Takasuga A., Setoguchi K., Pfuhl R., Flisikowski K., Fries R., Klopp N., Furbass R., Weikard R., Kuhn C. (2009). Dissection of genetic factors modulating fetal growth in cattle indicates a substantial role of the non-SMC condensin I complex, subunit G (NCAPG) gene. Genetics.

[18] Liu R., Sun Y., Zhao G., Wang F., Wu D., Zheng M., Chen J., Zhang L., Hu Y., Wen J. (2013). Genome-wide association study identifies Loci and candidate genes for body composition and meat quality traits in Beijing-You chickens. PLoS ONE.

[19] Makvandi-Nejad S., Hoffman G.E., Allen J.J., Chu E., Gu E., Chandler A.M., Loredo A.I., Bellone R.R., Mezey J.G., Brooks S.A. (2012). Four loci explain 83% of size variation in the horse. PLoS ONE.

[20] Rubin C.J., Megens H.J., Martinez B.A., Maqbool K., Sayyab S., Schwochow D., Wang C., Carlborg O., Jern P., Jorgensen C.B. (2012). Strong signatures of selection in the domestic pig genome. Proc. Natl. Acad. Sci. USA.

[21] Liu Y., Duan X., Chen S., He H., Liu X. (2015). NCAPG is differentially expressed during longissimus muscle development and is associated with growth traits in Chinese Qinchuan beef cattle. Genet. Mol. Biol..

[22] Sewda A., Agopian A.J., Goldmuntz E., Hakonarson H., Morrow B.E., Taylor D., Mitchell L.E. (2019). Gene-based genome-wide association studies and meta-analyses of conotruncal heart defects. PLoS ONE.

[23] Hu X., Xing Y., Fu X., Yang Q., Ren L., Wang Y., Li Q., Li J., Zhang L. (2020). NCAPG Dynamically Coordinates the Myogenesis of Fetal Bovine Tissue by Adjusting Chromatin Accessibility. Int. J. Mol. Sci..

[24] Han X., Jiang T., Yu L., Zeng C., Fan B., Liu B. (2012). Molecular characterization of the porcine MTPAP gene associated with meat quality traits: Chromosome localization, expression distribution, and transcriptional regulation. Mol. Cell. Biochem..

[25] Yang H., Pu L., Li R., Zhu R. (2023). NCAPG is transcriptionally regulated by CBX3 and activates the Wnt/beta-catenin signaling pathway to promote proliferation and the cell cycle and inhibit apoptosis in colorectal cancer. J. Gastrointest. Oncol..

[26] Hou J., Huang P., Xu M., Wang H., Shao Y., Weng X., Liu Y., Chang H., Zhang L., Cui H. (2023). Nonstructural maintenance of chromatin condensin I complex subunit G promotes the progression of glioblastoma by facilitating Poly (ADP-ribose) polymerase 1-mediated E2F1 transactivation. Neuro Oncol..

[27] Zhu Y., Tong H.L., Li S.F., Yan Y.Q. (2017). Effect of TCEA3 on the differentiation of bovine skeletal muscle satellite cells. Biochem. Biophys. Res. Commun..

[28] Zhen H., Shen J., Wang J., Luo Y., Hu J., Liu X., Li S., Hao Z., Li M., Shi B. (2022). Characteristics and Expression of circ_003628 and Its Promoted Effect on Proliferation and Differentiation of Skeletal Muscle Satellite Cells in Goats. Animals.

[29] Fong A.P., Tapscott S.J. (2013). Skeletal muscle programming and re-programming. Curr. Opin. Genet. Dev..

[30] Dej K.J., Ahn C., Orr-Weaver T.L. (2004). Mutations in the Drosophila condensin subunit dCAP-G: Defining the role of condensin for chromosome condensation in mitosis and gene expression in interphase. Genetics.

[31] Li P., Wen J., Ren X., Zhou Y., Xue Y., Yan Z., Li S., Tian H., Tang X.G., Zhang G.J. (2021). MicroRNA-23b-3p targets non-SMC condensing I complex subunit G to promote proliferation and inhibit apoptosis of colorectal cancer cells via regulation of the PI3K/AKT signaling pathway. Oncol. Lett..

[32] Listrat A., Lebret B., Louveau I., Astruc T., Bonnet M., Lefaucheur L., Picard B., Bugeon J. (2016). How Muscle Structure and Composition Influence Meat and Flesh Quality. Sci. World J..

[33] Huang C., Ge F., Ma X., Dai R., Dingkao R., Zhaxi Z., Burenchao G., Bao P., Wu X., Guo X. (2021). Comprehensive Analysis of mRNA, lncRNA, circRNA, and miRNA Expression Profiles and Their ceRNA Networks in the Longissimus Dorsi Muscle of Cattle-Yak and Yak. Front. Genet..

[34] Zeng Q., Du Z.Q. (2023). Advances in the discovery of genetic elements underlying longissimus dorsi muscle growth and development in the pig. Anim. Genet..

[35] Suzuki S., Yamanouchi K., Soeta C., Katakai Y., Harada R., Naito K., Tojo H. (2002). Skeletal muscle injury induces hepatocyte growth factor expression in spleen. Biochem. Biophys. Res. Commun..

[36] Alam M.Z., Haque M.A., Iqbal A., Lee Y.M., Ha J.J., Jin S., Park B., Kim N.Y., Won J.I., Kim J.J. (2023). Genome-Wide Association Study to Identify QTL for Carcass Traits in Korean Hanwoo Cattle. Animals.

[37] Zabidi M.A., Stark A. (2016). Regulatory Enhancer-Core-Promoter Communication via Transcription Factors and Cofactors. Trends Genet..

[38] Improda T., Morgera V., Vitale M., Chiariotti L., Passaro F., Feola A., Porcellini A., Cuomo M., Pezone A. (2023). Specific Methyl-CpG Configurations Define Cell Identity through Gene Expression Regulation. Int. J. Mol. Sci..

[39] Huang Y.Z., Zhang Z.J., He H., Cao X.K., Song C.C., Liu K.P., Lan X.Y., Lei C.Z., Qi X.L., Bai Y.Y. (2017). Correlation between ZBED6 Gene Upstream CpG Island methylation and mRNA expression in cattle. Anim. Biotechnol..

[40] Wei D., Li A., Zhao C., Wang H., Mei C., Khan R., Zan L. (2018). Transcriptional Regulation by CpG Sites Methylation in the Core Promoter Region of the Bovine SIX1 Gene: Roles of Histone H4 and E2F2. Int. J. Mol. Sci..

[41] Wu W., Ren Z., Liu H., Wang L., Huang R., Chen J., Zhang L., Li P., Xiong Y. (2013). Core promoter analysis of porcine Six1 gene and its regulation of the promoter activity by CpG methylation. Gene.

[42] Tisato V., Castiglione A., Ciorba A., Aimoni C., Silva J.A., Gallo I., D’Aversa E., Salvatori F., Bianchini C., Pelucchi S. (2023). LINE-1 global DNA methylation, iron homeostasis genes, sex and age in sudden sensorineural hearing loss (SSNHL). Hum. Genomics.

[43] Wang J., Huang Y., Xu J., Yue B., Wen Y., Wang X., Lei C., Chen H. (2022). Pleomorphic adenoma gene 1 (PLAG1) promotes proliferation and inhibits apoptosis of bovine primary myoblasts through the PI3K-Akt signaling pathway. J. Anim. Sci..

[44] Cui J.X., Gong Z.A., Zhang W.T., Liu K., Li T., Shao S.L., Zhang W.W. (2022). Effects of transcription factor SIX2 gene on the proliferation of bovine skeletal muscle satellite cells. Zhongguo Ying Yong Sheng Li Xue Za Zhi.

[45] Shi P., Ruan Y., Liu W., Sun J., Xu J., Xu H. (2023). Analysis of Promoter Methylation of the Bovine FOXO1 Gene and Its Effect on Proliferation and Differentiation of Myoblasts. Animals.

[46] Zhou D., Xu H., Chen W., Wang Y., Zhang M., Yang T. (2018). Study on the transcriptional regulatory mechanism of the MyoD1 gene in Guanling bovine. Rsc Adv..

[47] Wei D., Raza S.H.A., Wang X., Khan R., Lei Z., Zhang G., Zhang J., Luoreng Z., Ma Y., Alamoudi M.O. (2022). Tissue Expression Analysis, Cloning, and Characterization of the 5’-Regulatory Region of the Bovine LATS1 Gene. Front. Vet. Sci..

[48] Du X.H., Gan Q.F., Yuan Z.R., Gao X., Zhang L.P., Gao H.J., Li J.Y., Xu S.Z. (2013). Polymorphism of MyoD1 and Myf6 genes and associations with carcass and meat quality traits in beef cattle. Genet. Mol. Res..

[49] Gellhaus B., Böker K.O., Gsaenger M., Rodenwaldt E., Hüser M.A., Schilling A.F., Saul D. (2023). Foxo3 Knockdown Mediates Decline of Myod1 and Myog Reducing Myoblast Conversion to Myotubes. Cells.

[50] Zhou D., Wang Y., Yang R., Wang F., Zhao Z., Wang X., Xie L., Tian X., Wang G., Li B. (2022). The MyoD1 Promoted Muscle Differentiation and Generation by Activating CCND2 in Guanling Cattle. Animals.

[51] Blum R., Dynlacht B.D. (2013). The role of MyoD1 and histone modifications in the activation of muscle enhancers. Epigenetics.

[52] Lee E.A., Kim J.M., Lim K.S., Ryu Y.C., Jeon W.M., Hong K.C. (2012). Effects of variation in porcine MYOD1 gene on muscle fiber characteristics, lean meat production, and meat quality traits. Meat Sci..

[53] Sun J., Ruan Y., Xu J., Shi P., Xu H. (2023). Effect of Bovine MEF2A Gene Expression on Proliferation and Apoptosis of Myoblast Cells. Genes.

[54] Tizioto P.C., Coutinho L.L., Mourão G.B., Gasparin G., Malagó-Jr W., Bressani F.A., Tullio R.R., Nassu R.T., Taylor J.F., Regitano L.C.A. (2016). Variation in myogenic differentiation 1 mRNA abundance is associated with beef tenderness in Nelore cattle. Anim. Genet..

[55] Ortega-Martínez S. (2015). A new perspective on the role of the CREB family of transcription factors in memory consolidation via adult hippocampal neurogenesis. Front. Mol. Neurosci..

[56] Montminy M., Koo S.H., Zhang X. (2004). The CREB Family: Key regulators of hepatic metabolism. Ann Endocrinol..

[57] Li G., Jiang Q., Xu K. (2019). CREB family: A significant role in liver fibrosis. Biochimie.

[58] Feng Y., Raza S.H.A., Liang C., Wang X., Wang J., Zhang W., Zan L. (2022). CREB1 promotes proliferation and differentiation by mediating the transcription of CCNA2 and MYOG in bovine myoblasts. Int. J. Biol. Macromol..

[59] Kim N.H., Sung N.J., Shin S., Ryu D., Youn H., Park S. (2021). Sauchinone inhibits the proliferation, migration and invasion of breast cancer cells by suppressing Akt-CREB-MMP13 signaling pathway. Bioscience Rep..

[60] Guo L., Yin M., Wang Y. (2018). CREB1, a direct target of miR-122, promotes cell proliferation and invasion in bladder cancer. Oncol. Lett..

[61] Tooley J.G., Catlin J.P., Schaner Tooley C.E. (2021). CREB-mediated transcriptional activation of NRMT1 drives muscle differentiation. Transcription.

[62] Erdenee S., Akhatayeva Z., Pan C., Cai Y., Xu H., Chen H., Lan X. (2021). An insertion/deletion within the CREB1 gene identified using the RNA-sequencing is associated with sheep body morphometric traits. Gene.

[63] Xiong Z., Wang M., You S., Chen X., Lin J., Wu J., Shi X. (2022). Transcription Regulation of Tceal7 by the Triple Complex of Mef2c, Creb1 and Myod. Biology.

[64] Sugasawa T., Tome Y., Takeuchi Y., Yoshida Y., Yahagi N., Sharma R., Aita Y., Ueda H., Maruyama R., Takeuchi K. (2020). Influence of Intermittent Cold Stimulations on CREB and Its Targeting Genes in Muscle: Investigations into Molecular Mechanisms of Local Cryotherapy. Int. J. Mol. Sci..

[65] Bo Y.Y., Liang L.D., Hua Y.J., Zhao Z., Yao M.S., Shan L.B., Liang C.Z. (2021). High-purity DNA extraction from animal tissue using picking in the TRIzol-based method. Biotechniques.

[66] Salvatori F., D’Aversa E., Serino M.L., Singh A.V., Secchiero P., Zauli G., Tisato V., Gemmati D. (2023). miRNAs Epigenetic Tuning of Wall Remodeling in the Early Phase after Myocardial Infarction: A Novel Epidrug Approach. Int. J. Mol. Sci..

[67] Zhang H.M., Chen H., Liu W., Liu H., Gong J., Wang H., Guo A.Y. (2012). AnimalTFDB: A comprehensive animal transcription factor database. Nucleic Acids Res..

[68] Hall B.G. (2013). Building phylogenetic trees from molecular data with MEGA. Mol. Biol. Evol..

