# Supplementary material for: Unlocking the Transcriptional Control of NCAPG in Bovine Myoblasts: CREB1 and MYOD1 as Key Players
Source: Int J Mol Sci. 2024 Feb 21;25(5):2506. doi: 10.3390/ijms25052506 (PMC10931257; doi:10.3390/ijms25052506)
Supplement: Supplementary file 1 [file ijms-25-02506-s001.zip › Figure S2.Agarose gel electrophoresis of the original deletion promoter fragment..pdf]

Supplementary Materials:

A

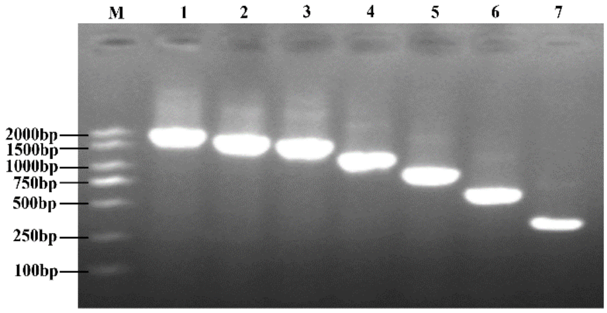

B

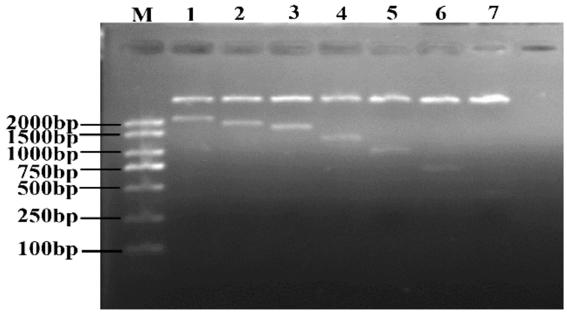

Figure S2. Agarose gel electrophoresis of the original deletion promoter fragment. (A) Different lengths of fragments of NCAPG promoter cloned by PCR. (B) Detection of vectors construction of NCAPG promoter in different lengths fragments. M: marker, 1: NCAPG-F1(-1952/+87), 2: NCAPG-F2(-1686/+87), 3: NCAPG-F3(-1519/+87), 4: NCAPG-F4(-1151/+87), 5: NCAPG-F5(-875/+87), 6: NCAPG-F6(-598/+87), and 7: NCAPG-F7(-350/+87).
