# Supplementary material for: Unlocking the Transcriptional Control of NCAPG in Bovine Myoblasts: CREB1 and MYOD1 as Key Players
Source: Int J Mol Sci. 2024 Feb 21;25(5):2506. doi: 10.3390/ijms25052506 (PMC10931257; doi:10.3390/ijms25052506)
Supplement: Supplementary file 1 [file ijms-25-02506-s001.zip › Table S1.pdf]

Supplementary Information

Supplementary Table S1. The primer sequences for RT-PCR, qRT-PCR, Promoter cloning, site-mut, EMSA and Overexpression

Item	Primer name	Primer Sequence(5′ to 3′)
RT-PCR	*NCAPG*	F: TCTTCCAACTCTGCAAACACTG
		R: GCCTGAGGATTTAACCCACTT
	CREB1	F: TGTTATGGCGTCTTCCCCAG
		R: TTCAAGCACTGCCACTCTGT
	MYOD1	F: CGCTCCCTTCCCAAGTGTAT
		R: TACAAAGTCCCTGTCGCACC
	MYOC	F: TACAGCAACCTCGTCCGTC
		R: AATTCCATTTAGCAACTTCCCT
	Pax7	F: AGGTCTGGTTCAGTAACCGC
		R: GCTGACGGGGTTCATGTGAT
	MHC	F: TGCAACCATGAGTGCGAGT
		R: CAGAGGGCTTACACAAGGGG
	CDK2	F: AGGGAACGTACGGAGTTGTG
		R: GACATCCAGCAGCTTGACAAT
	PCNA	F: CCTTGGTGCAGCTAACCCTT
		R: TTGGACATGCTGGTGAGGTT
	CyclinD1	F: ATGAAGGAGACCATCCCCCT
		R: CGCCAGGTTCCACTTGAGTT
	GAPDH	F: AAGTTCAACGGCACAGTCA
		R: GTCATAAGTCCCTCCACGAT
Promoter cloning	*NCAPG F/R*	F: GCAAGGGAATGGATGAAC
		R: GAAGGCCTCCTTAATCAG
	NCAPG-F1	F: CTAGCTAGCTCGGCTCCAATAGTCGAATC
	NCAPG-F2	F: CTAGCTAGCGTGGGCATTGACCAGAAG
	NCAPG-F3	F:CTAGCTAGCATCCCGTGGAGGAGGAAATG
	NCAPG-F4	F: CTAGCTAGCGGAGGGAAGATAGTTCGATAGG
	NCAPG-F5	F: CTAGCTAGCACCTCTATAGGGCAGAAC
	NCAPG-F6	F:CTAGCTAGCAGCTGCACTAATCAGACC
	NCAPG-F7	F: CTAGCTAGCGCAAGGGAATGGATGAAC
	NCAPG	R:CCGCTCGAGGAAGGCCTCCTTAATCAG
Site-mut and EMSA	*MYOD1*	F:AAGACCCGATTAGGCCTAGGCGGTGGTCGTCGGCTCCAATAGTCGAATC
	MYOD1	R: GATTCGACTATTGGAGCCGAGCGGTGGTCGCTAGGCCTAATCGGGTCTT
	mMYOD1	F: CCAGCCGCAAGACCCGATTAGGCCTAGGCGGTGGTCGTCGGCTCCAATAGTCGAATCTC
	mMYOD1	R: GAGATTCGACTATTGGAGCCGACGACCACCGCCTAGGCCTAATCGGGTCTTGCGGCTGG
	CREB1	F: ATGTTGAAGGCGGTGGATTCAAGAGAAGGTCACTCGCCGCTCTACGCACTCGC
	CREB1	R: GCGAGTGCGTAGAGCGGCGAGAGAGAAGGTCATGAATCCACCGCCTTCAACAT
	mCREB1	F: CGATGTTGAAGGCGGTGGATTCATGAGAAGGTCGACTCGCCGCTCTACGCACTCGCGGT
	mCREB1	R: ACCGCGAGTGCGTAGAGCGGCGAGTCGACCTTCTCATGAATCCACCGCCTTCAACATCG
	NCAPG	F: AAGGCGGCCGCTATGGGGAAGGAGAAGAGACTGC
		R: CCCTCTAGACTAACTTGTATCTTCATTGAGAAATTGTGCA
Overexpression	*MYOD1*	F: CGCGGATCCATGGAGCTACTGTCGCCACC
		R: CCGCTCGAGGAGCACCTGGTATATCGGGTT
	CREB1	F: CGCGGATCCATGACCATGGAATCTGGAGCCGAGA
		R: GCTCTAGAATCTGATTTGTGGCAGTAAAGGT
