# Supplementary figures and images for: Unlocking the Transcriptional Control of NCAPG in Bovine Myoblasts: CREB1 and MYOD1 as Key Players
Source: Int J Mol Sci. 2024 Feb 21;25(5):2506. doi: 10.3390/ijms25052506 (PMC10931257; doi:10.3390/ijms25052506)

## Supplementary Materials:

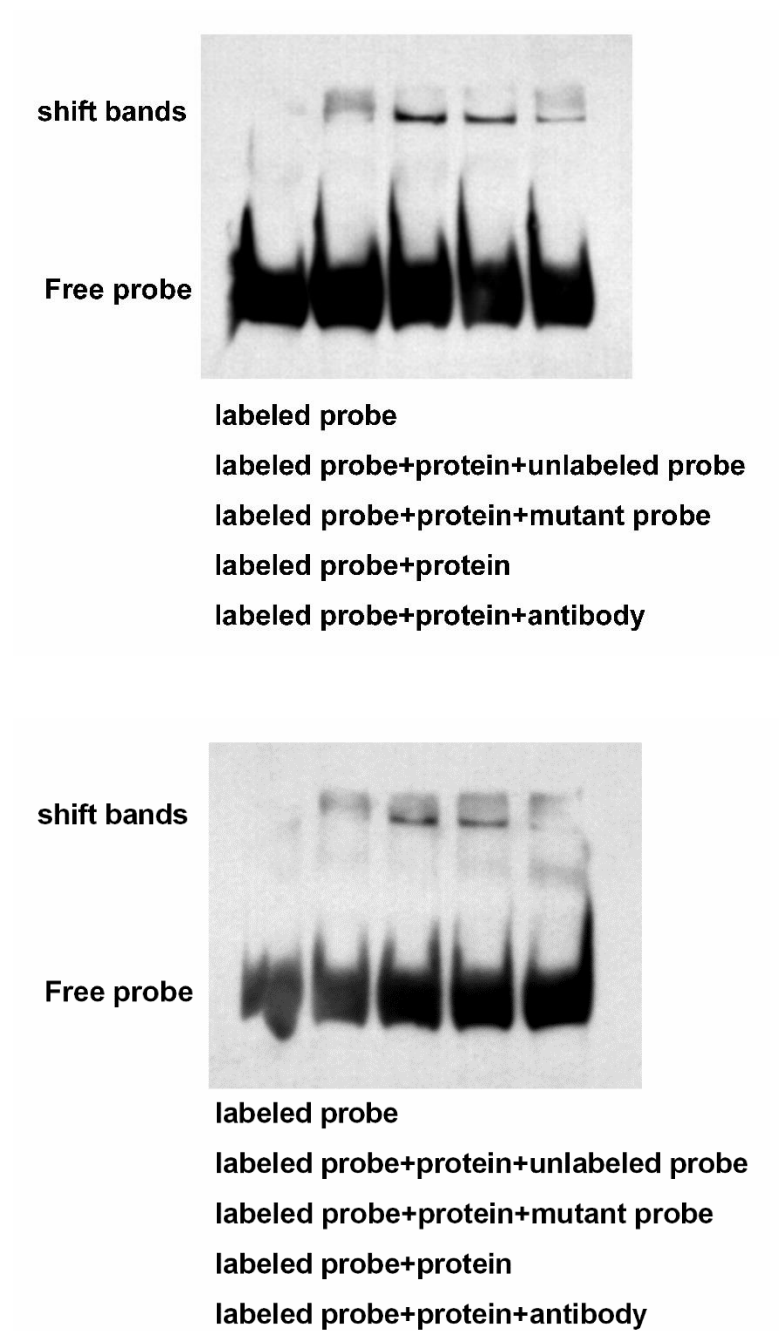

Figure S1. Original EMSA figures

Supplement: Supplementary file 1 [file ijms-25-02506-s001.zip › Figure S1. Original EMSA figures.pdf]
